# Visual cortex in aging and Alzheimer's disease: changes in visual field maps and population receptive fields

**DOI:** 10.3389/fpsyg.2014.00074

**Published:** 2014-02-07

**Authors:** Alyssa A. Brewer, Brian Barton

**Affiliations:** Laboratory of Visual Neuroscience, Department of Cognitive Sciences, Center for Cognitive Neuroscience, University of CaliforniaIrvine, CA, USA

**Keywords:** aging, Alzheimer's disease, vision, visual field mapping, population receptive field modeling

## Abstract

Although several studies have suggested that cortical alterations underlie such age-related visual deficits as decreased acuity, little is known about what changes actually occur in visual cortex during healthy aging. Two recent studies showed changes in primary visual cortex (V1) during normal aging; however, no studies have characterized the effects of aging on visual cortex beyond V1, important measurements both for understanding the aging process and for comparison to changes in age-related diseases. Similarly, there is almost no information about changes in visual cortex in Alzheimer's disease (AD), the most common form of dementia. Because visual deficits are often reported as one of the first symptoms of AD, measurements of such changes in the visual cortex of AD patients might improve our understanding of how the visual system is affected by neurodegeneration as well as aid early detection, accurate diagnosis and timely treatment of AD. Here we use fMRI to first compare the visual field map (VFM) organization and population receptive fields (pRFs) between young adults and healthy aging subjects for occipital VFMs V1, V2, V3, and hV4. Healthy aging subjects do not show major VFM organizational deficits, but do have reduced surface area and increased pRF sizes in the foveal representations of V1, V2, and hV4 relative to healthy young control subjects. These measurements are consistent with behavioral deficits seen in healthy aging. We then demonstrate the feasibility and first characterization of these measurements in two patients with mild AD, which reveal potential changes in visual cortex as part of the pathophysiology of AD. Our data aid in our understanding of the changes in the visual processing pathways in normal aging and provide the foundation for future research into earlier and more definitive detection of AD.

## Introduction

In order to carefully evaluate alterations of visual cortex in age-related neurodegenerative diseases such as Alzheimer's disease (AD), we must first have a clear understanding of what changes occur across visual cortex during healthy aging (Jackson and Owsley, 2003; Yankner et al., 2008). Studies of the aging visual cortex are limited, however. Although the organization and function of early visual cortex (i.e., V1, V2, V3, hV4) have been well characterized in human, these measurements have almost exclusively been in healthy young adults or specific patient populations (Sereno et al., 1995; Deyoe et al., 1996; Dougherty et al., 2003; Crossland et al., 2008; Baseler et al., 2011; Brewer and Barton, 2012b).

Two recent neuroimaging studies have examined V1 in healthy aging subjects. Using functional magnetic resonance imaging (fMRI) with traveling wave visual field mapping (VFM) methods, Crossland et al. (2008) showed that primary visual cortex (V1) of healthy aging subjects has lower blood-oxygen-level-dependent (BOLD) activity in the fovea compared to healthy young adults. This study also demonstrated that aging has no effect on fixation stability, which is important to demonstrate the feasibility of VFM measurements in this subject population. Building on these results, we (Brewer and Barton, 2012a) used fMRI population receptive field (pRF) modeling methods (Dumoulin and Wandell, 2008) to demonstrate several changes in the V1 of aging subjects compared to young subjects, including (1) a decrease in surface area of foveal V1 from 0 to 3°, and (2) an increase in the pRF sizes within this same foveal region. These alterations may account for behavioral changes associated with vision in aging such as reduced visual acuity and decreased contrast sensitivity at medium and high spatial frequencies (Elliott, 1987; Whitaker and Elliott, 1992). In addition, these previous neuroimaging findings may also reflect anatomical changes in the aging visual pathways, such as a decline in the retinal nerve fiber layer thickness (Balazsi et al., 1984; Parikh et al., 2007) and a loss of retinal photoreceptors (Gao and Hollyfield, 1992; Curcio et al., 1993; Jackson et al., 2002).

These measurements have demonstrated several changes in healthy aging just within primary visual cortex, but very little is known about the effects of aging on the structural and functional characteristics of VFMs beyond V1. The present measurements are the first to expand our fledgling understanding of the effects of normal aging beyond primary visual cortex.

Once we can distinguish the cortical changes related to healthy aging from those resulting from the pathophysiology of the disease, we can begin to investigate changes in visual cortex in age-related disorders like AD. AD, the most common form of dementia, is characterized by progressive cognitive deficits including disturbances in memory, language, executive function, and vision (Black, 1996; Yankner et al., 2008). Early detection and accurate diagnosis are key in the hope for a cure for dementias such as AD, as early, accurate diagnosis would allow for more timely initiation of treatments. As visual symptoms can occur early in AD, it is possible that measurements of changes in visual cortex in these patients could aid early detection of neurodegeneration. Cortical representations of visual space (e.g., VFMs) in particular provide a highly-structured functional measurement that might be used to detect subtle effects of neurodegeneration early in the disease process. A better understanding of the progression of the pathology within visual cortex could also help to target drug research for therapeutic interventions (Rosen, 2004).

AD can present with a variable range of visual symptoms across subjects, from lower level deficits such as changes in visual field coverage, contrast sensitivity, color discrimination, visuospatial perception, and visual processing speed (Cronin-Golomb et al., 1993; Chan et al., 2001; Jackson and Owsley, 2003; Mapstone et al., 2003; Sauer et al., 2006; Thiyagesh et al., 2009) to higher level deficits such as problems in visual attention and in feature recognition of complex objects such as faces (Parasuraman et al., 1992; Giannakopoulos et al., 1999, 2000; Holroyd and Shepherd, 2001; Pache et al., 2003; Tang-Wai et al., 2004; van Rhijn et al., 2004; Bokde et al., 2006; Thiyagesh et al., 2009). These deficits could be attributed perhaps to a random pattern of neurodegeneration across regions of visual cortex (Jackson and Owsley, 2003). However, there is also emerging evidence for a more precise distribution of neurodegeneration in the AD visual pathways, with some studies showing neurofibrillary tangles and neuritic senile plaques increasing steadily from primary to associative visual cortex (Lewis et al., 1987; Black, 1996; Giannakopoulos et al., 1999; Yankner et al., 2008). In addition, several studies have found that AD patients have widespread axonal degeneration of the optic nerves and a reduction of retinal ganglion cells (Hinton et al., 1986; Danesh-Meyer et al., 2006; Iseri et al., 2006; Berisha et al., 2007; Paquet et al., 2007). These cortical and retinal lesions both result in the disruption of normal inputs to the visual processing streams, which we expect to be reflected in changes in the organization, functionality and connectivity of visual cortex. However, despite many descriptions of visual symptoms in AD, little is known about the extent of changes in visual cortex that underlie these visual deficits.

Here we use pRF modeling (Dumoulin and Wandell, 2008) to compare detailed structural and functional measurements of occipital VFMs V1, V2, V3, and hV4 between healthy young adults and normally aging subjects. We also demonstrate the feasibility of these measurements in patients with early, mild AD and present the first characterization of VFMs V1, V2, V3, and hV4 in these patients.

## Methods

### Subject recruitment and characterization

Eleven subjects were recruited for this study: five healthy young adult subjects aged 24–36 years (two female; mean age = 28 years, *SD* = 4.8 years), four healthy, normally aging subjects aged 57–70 years (three female; mean age = 63.5 years, *SD* = 5.4 years), and two patients diagnosed with early, mild probable AD aged 70 (AD-S11; female) and 72 (AD-S10; male) years (mean age = 71 years, *SD* = 1.4 years). There was no significant difference between the ages of the healthy aging and AD subject groups, as determined by a two-tailed, independent samples *t*-test [*t*_(4)_ = 1.816; *p* = 0.14]. The young adult subjects were recruited from the students and faculty at the University of California, Irvine (UCI). The aging and AD subjects were recruited from the normally aging and AD cohorts enrolled at the Alzheimer's Disease Research Center (ADRC) at UCI. Subjects are included in each cohort based upon neuropsychiatric testing and clinical diagnosis through the ADRC, with a normal Mini-Mental State Exam (MMSE) (Folstein et al., 1975) score between 29 and 30 for healthy aging subjects and between 21 and 26 for subjects with a diagnosis of mild probable AD. AD subjects were included in the study with a diagnosis of mild probable AD within the last year. ADRC cohorts undergo a battery of tests and longitudinal data collection including: demographics, medical history, medications, family history, physical exam, neurological exam, cognitive testing and diagnosis, APOE genotypes, and comprehensive neuropsychological testing. Neuropsychological tests are scored by ADRC expert raters and clinicians. Clinical diagnosis is confirmed by consensus among two or more ADRC physicians. All subjects recruited for this study had no history of previous head injury, alcoholic brain damage, a pre-existing visual disorder, or any additional significant physical or psychiatric conditions. All subjects had normal or corrected-to-normal visual acuity (at least 20/25) with no underlying visual disease. The Institutional Review Board at UCI approved all aspects of the experimental protocol. Informed consent was obtained from all subjects prior to the initiation of any experiments.

### Experimental design

Each subject underwent 1–2 fMRI scan sessions, in which one high-resolution, T1-weighted anatomical volume, one T1-weighted in-plane anatomical scan, and 8 functional VFM scans were collected. Visual stimuli were generated using the Psychophysics Toolbox (Brainard, 1997; Pelli, 1997) in the Matlab programming environment on a Dell Optiplex desktop. Stimuli were back-projected via a Christie DLV1400-DX DLP projector onto a screen at the head end of the bore of the magnet (spatial resolution: 1024 × 768 pixels, refresh rate: 60 Hz).

All subjects were shown the stimulus display and experiment room setup prior to starting the experiment. Particular time was spent with the AD subjects in order to ensure their abilities to comply with the experimental task throughout each scan. Subjects performed several trials of the stimulus on a display in the scanner control room. During this practice, they were shown the stimulus characteristics, practiced fixating on the center cross, and practiced attending to the movements of the checkerboard pattern. All subjects were able to perform these tasks well prior to starting data collection within the scanner.

For the scans, subjects lay supine in the bore of the magnet and viewed the display on an angled front surface mirror mounted on the head coil (viewing distance = ~70 cm). Head movements were minimized with padding and tape. Subjects were required to maintain fixation on a central cross for the duration of a single scan; regular blinking was encouraged. Each scan was ~3 min in length, with short breaks between each scan. Subjects were reminded to fixate and to perform the attentional task between each 3 min scan. Eye position at fixation was verified using an MR-compatible long range remote eye tracking system (*Applied Science Laboratories*, Bedford, MA). All subjects were able to maintain fixation throughout all scans. No effects of problems with eye position (e.g., consistent offset, nystagmus) could explain the results presented below (for models of poor fixation, see Baseler et al., 2002; Crossland et al., 2008; Levin et al., 2010).

### Moving bar stimulus for visual field mapping

During all functional scans, the subject viewed a moving bar stimulus comprised of high-contrast, flickering, black, and white checkerboard patterns similar to the pattern used in typical wedge and ring traveling wave stimuli. The checkerboard pattern reversed contrast at a temporal frequency of 2 Hz, producing checkerboard rows that appeared to be moving in the opposite direction to adjacent rows (Brewer and Barton, 2012b). The stimulus subtended a maximum radius of 11° of visual angle. The moving bar was displaced in discrete steps every 2 s in synchrony with the fMRI volume acquisition and moved across the visible screen in eight different configurations (four orientations: 0, 45, 90, and 135° from vertical with two orthogonal motion directions) for a total presentation time of 192 s at one cycle/scan. Four mean-luminance (“blank”) periods for use in the pRF analysis (Dumoulin and Wandell, 2008) were inserted in the last 12 s of each 48 s period, at a frequency of four cycles/scan (a non-stimulus frequency).

Subjects maintained fixation on one of two large fixation crosses, spanning either the diagonals from the corners of the field of view (“**X**”) or the midpoints of each of the sides of the field of view (“+”). The lines of each fixation cross were roughly 0.5° wide, and they randomly switched between the two screen positions every 2–4 s during the progression of the bar stimulus across the visual field. Subjects were instructed to attend to the moving bar stimulus and were required to respond with a button press (not in sync with the visual stimulus position changes or mean-luminance periods) to an intermittent, subtle change in the motion direction of the checkerboard pattern.

### Anatomical data acquisition and processing

**Figure 1:**
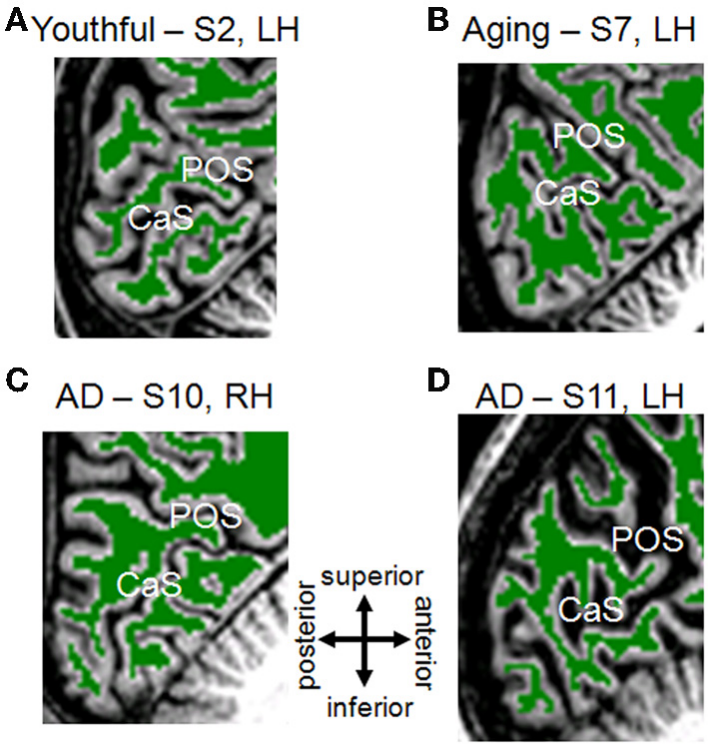
**White/Gray matter segmentation for young, healthy aging, and mild Alzheimer's disease subjects**. Each panel is a T1-weighted 3D MPRAGE image showing a sagittal slice near the midline of the brain, cropped to the occipital lobe with the calcarine sulcus (CaS; home to V1) and the parietal-occipital sulcus (POS) visible. Green-colored overlay represents white matter identified by an automated algorithm (Teo et al., 1997) and adjusted by hand-editing to minimize segmentation errors (Dougherty et al., 2003). Gray regions are gray matter, and white regions are white matter not segmented in the cerebellum. Gray matter is grown from the boundary of the white matter and bounded by the cerebral spinal fluid (CSF; black adjacent to gray matter). Portions of skull and dura mater are also visible. **(A)** Youthful Subject 2, left hemisphere. **(B)** Aging Subject 7, left hemisphere. **(C)** Mild Alzheimer's disease Subject 10, right hemisphere. **(D)** Mild Alzheimer's disease Subject 11, left hemisphere. Compare AD-S10's intact anatomy to the visibly altered visual field maps from this subject in Figure 2. Note also the reverse pattern for AD-S11, who has grossly intact map sizes, but more strikingly increased CSF-filled space.

Scanning was conducted on the 3T Philips Achieva MR scanner at UCI with an eight channel SENSE imaging head coil. One high-resolution, whole-brain anatomical data set was acquired for each subject (T1-weighted 3D MPRAGE, 1 mm^3^ voxels, *TR* = 8.4 ms, *TE* = 3.7 ms, flip = 8°, SENSE factor = 2.4). There were no problems with T1 acquisition (e.g., due to subject motion) for any scans. Custom Matlab-based software (*mrGray* in the *mrVista* package, freely available online at http://white.stanford.edu/software) was used to define the white matter in the structural anatomical image for each individual subject (Teo et al., 1997). This software uses an automated algorithm to initially select the white matter, followed by hand-editing to minimize segmentation errors (Dougherty et al., 2003). Hand-editing allows for careful, accurate segmentation of cortex not only in individual subjects with normal anatomy, but also in individuals with unusual and/or abnormal anatomy, such as is expected in aging and AD subjects (Figure 1). Gray matter was grown from the segmented white matter to form a layer covering the white matter surface. The cortical surface was then represented as a mesh at the white/gray-matter border, which was used to render a smooth 3D cortical surface or to flatten the cortical representation, with light gray regions indicating gyri and dark gray regions representing sulci (Wandell et al., 2000).

In addition, one anatomical in-plane image was acquired before each set of functional scans, with the same slice prescription as the functional scans, but with a higher spatial resolution (1 × 1 × 3 mm voxels). These T1-weighted slices were physically in register with the functional slices and were used to align the functional data with the high-resolution anatomical data, first by a manual co-registration and then by a semi-automated 3D co-registration algorithm, a mutual information method (Maes et al., 1997; Nestares and Heeger, 2000).

### Functional data acquisition and processing

Functional MR data for VFM measurements were acquired on the same scanner as the anatomical data, with ~35 oblique slices oriented close to parallel to the calcarine sulcus (T2-weighted, gradient echo imaging, *TR* = 2 s, *TE* = 30 ms, flip = 90°, SENSE factor = 1.7, reconstructed voxel size of 1.875 × 1.875 × 3 mm, no gap). For each subject, data in each fMRI session were analyzed voxel-by-voxel with no spatial smoothing, using the same custom Matlab-based software package (*mrVista*; http://white.stanford.edu/software). Head movements across scans were examined by comparing the mean value maps of the BOLD signals. Because all scans had less than one voxel of head motion, no motion correction algorithm was applied here. The BOLD time series from each scan was high-pass filtered to remove low-frequency sources of physiological noise and averaged together to form one mean time series for each subject, which was then used in the pRF model analysis (Dumoulin and Wandell, 2008).

### Population receptive field modeling analysis

pRF modeling is an emerging method for detailed VFM experiments (Dumoulin and Wandell, 2008; Amano et al., 2009; Baseler et al., 2011; Harvey and Dumoulin, 2011; Haak et al., 2012a,b; Zuiderbaan et al., 2012; Harvey et al., 2013) that can estimate cortical visual field responses to any stimulus that periodically covers visual space, such as the moving bars used here. Briefly, in retinotopically-organized regions of visual cortex, each voxel in a VFM contains a population of neurons with similar receptive fields (RFs). We call the average RF measured across a voxel a “population receptive field” (pRF). Thus, pRF modeling treats each voxel in a VFM as a pRF with a preferred center (x, y) and spread (σ). To determine this, the model creates a bank of 2D Gaussian pRFs of numerous possible sizes and visual field locations spanning the field of view, convolves each predicted response to the presented stimulus with the hemodynamic response function (HRF) (Boynton et al., 1996; Friston et al., 1998), and tests the result against the data. The pRF which best matches the data is then used to determine that voxel's variance explained, visual field representation, and pRF size. This method not only replicates results of VFM measurements from the travelling wave method in a model-based way, but allows for additional measures such as pRF sizes. Complete details of the pRF model analysis are described in Dumoulin and Wandell (2008).

**Figure 2:**
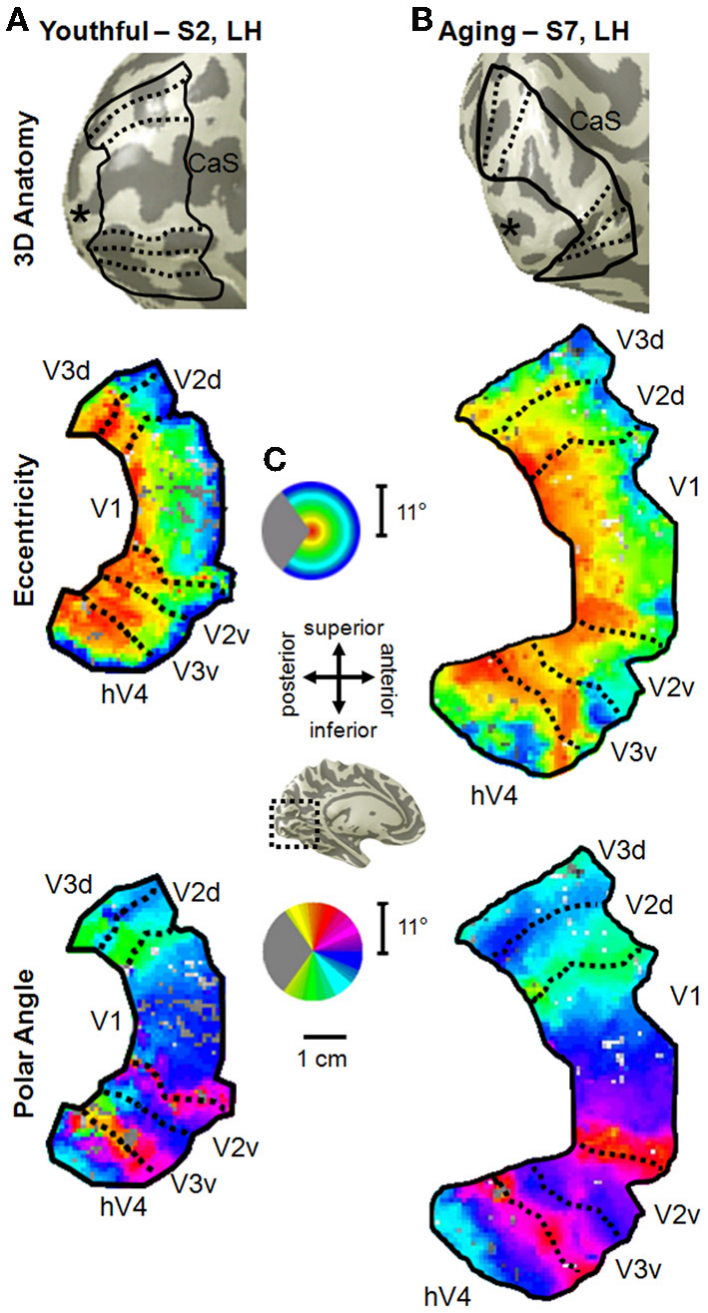
**Visual field map measurements in healthy young and aging subjects**. **(A)** Example of VFMs V1, V2, V3, and hV4 from a healthy young adult (S2, left hemisphere). VFM boundaries are overlaid on a 3D representation of S2's left hemisphere (top panel). The “^*^” denotes the occipital pole. A cropped, close-up view of the flattened cortical surface surrounding the calcarine sulcus is shown for measurements of eccentricity and polar angle in the middle and bottom panels, respectively. The pseudo-color overlay on each flattened cortical rendering represents the position in visual space that produces the strongest response at each cortical location [see colored legend insets in **(C)**]. The stimuli covered the central 11° radius of visual space. For clarity, the visual responses are only shown for the VFMs of interest, V1, V2, V3, hV4. **(B)** Example of VFMs V1, V2, V3, and hV4 from a healthy aging subject (S7, left hemisphere). Note regular, organized, orthogonal VFMs as seen in **(A)**. **(C)** Legends and scale bar for flattened cortical maps are shown. Approximate cortical location is depicted on an example left hemisphere 3D rendering (black dotted lines, central inset).

Here we used the pRF method to estimate VFMs and pRFs for posterior occipital VFMs V1, V2, V3, and hV4. Eccentricity {1} and angle [tan^−1^(*y*/*x*)] were derived from the 2D Gaussian models and plotted on the unfolded cortical surface for each hemisphere in each subject. pRF modeling uses percent variance explained as a primary measurement of the goodness-of-fit of the model to the BOLD time series data; here we independently assign each voxel a value for variance explained. Only voxels with variance explained ≥0.04 (corresponding to the standardly-used coherence threshold in traveling wave studies of 0.20) were assigned a phase corresponding to that voxel's peak response to the stimuli presented and considered for further analysis. We have measured the noise in visual cortex of all our subject groups using baseline measurements in early visual cortex with a combination of approaches, including photopic and scotopic visual stimuli (bars, wedges, rings) with traveling wave and pRF modeling methods. Our measurements show a maximum baseline noise level for coherence (from traveling wave measurements) of 0.15 and for variance explained (from pRF modeling measurements) of 0.03. The majority of our measurements presented here are well above these values (Figures 2, 6, 7).

Finally, it is important to note that the pRF modeling method allows for the use of the single moving bar stimulus to determine both the polar angle and eccentricity dimensions of the cortical representations of visual space within a single scan. This means that alterations seen in one dimension (e.g., eccentricity) that are not observed in the second dimension (e.g., polar angle) cannot be simply attributed to a problem with that particular scan.

### Visual field map definition

We define VFMs by the following criteria: (1) both a polar angle and an eccentricity gradient must be present, (2) the polar angle and eccentricity gradients must be orthogonal to one another, and (3) each VFM must represent a complete contralateral hemifield of visual space (e.g., Wandell et al., 2007; Brewer and Barton, 2012b). Here, as usual, the boundaries between V1, V2, V3, and hV4 were determined by manually tracing the polar angle reversals along the medial occipital wall and along the periphery of the eccentricity gradient representing the 11° radius stimulus. Because hemodynamic changes have been shown to occur with normal aging, we identified the phases at which boundary reversals occurred in order to compare the correct stimulus location in visual space with the corresponding cortical responses (D'Esposito et al., 1999, 2003; Crossland et al., 2008).

### Statistical analysis

For the following comparisons between young adult and healthy aging subjects of the measurements of VFM surface areas, variance explained, and pRF sizes, we divided up the eccentricity representation in each map in each hemisphere of each subject into specific bands. For each VFM, we created 10 regions of interest (ROIs) spanning 1° of visual angle along the eccentricity gradient from 0 to 10°, centered on every half degree (Figures 4–9). Each measurement was drawn from these 10 eccentricity-band ROIs for each subject, averaged between hemispheres for each subject, and then analyzed across subjects within each group. Two previous fMRI VFM studies comparing youthful and normal aging subjects indicated that the central 3° of visual angle about fixation is the most likely area to be affected by aging in V1. Aging subjects' V1 maps have significantly less surface area and larger pRF sizes in the central 3° relative to youthful subjects (Crossland et al., 2008; Brewer and Barton, 2012a). These neuroimaging results may reflect cortical changes related to behavioral measures of decreased visual acuity in normal aging (Elliott, 1987; Whitaker and Elliott, 1992). The same subjects are analyzed here as in the previous Brewer and Barton (Brewer and Barton, 2012a) study; however, we use a different statistical approach, and we present novel measurements of V2, V3, and hV4. Here, we perform one multivariate analysis of variance (MANOVA) for the central 3° and peripheral 3–10° of V1, V2, V3, and hV4 for each of the three measurements (surface area, variance explained, and pRF size).

## Results

### Comparisons between healthy aging and youthful subjects

#### Aging vs. youth: overall visual field map organization

For the youthful and healthy aging subjects, we were able to easily define the boundaries of V1, V2, V3, and hV4 in all subjects (Figure 2). All four VFMs in all hemispheres contained a complete representation of the contralateral hemifield, in line with previous measurements of these VFMs in young subjects (e.g., Engel et al., 1994, 1997; Sereno et al., 1995; Black, 1996; Brewer et al., 2005; Wandell et al., 2007; Brewer and Barton, 2012b) and in V1 in healthy aging subjects (Crossland et al., 2008; Brewer and Barton, 2012a). Note that human V2 and V3 are standardly considered in each hemisphere to be VFMs that represent complete hemifields of visual space with non-contiguous quarterfields (e.g., Brewer et al., 2002; Wandell et al., 2007).

In all hemispheres of these two subject groups, the confluent eccentricity gradient of the posterior VFMs corresponding to the 11° radius of our stimulus spanned from the foveal representation (Figure 2, *middle panel*, eccentricity—red/orange) on the occipital pole along calcarine to the more anterior peripheral representation (Figure 2, *middle panel*, eccentricity—green/blue/purple). The second dimension of visual space necessary to separate the confluent eccentricity representation of the occipital pole into specific VFMs is the polar angle. The polar angle gradient of V1 spanned the medial surface of the occipital pole from the dorsal edge of the calcarine sulcus on the cuneus (Figure 2, *bottom panel*, polar angle—cyan) to the ventral edge of the calcarine sulcus on the lingual gyrus (Figure 2, *bottom panel*, polar angle—magenta), with the lower vertical meridian of visual space represented dorsally and vice versa, as expected from our previous V1 measurements in these young and aging subjects (Brewer and Barton, 2012a). The dorsal lower vertical meridian representation of V1 reversed into V2d along the dorsal edge of the calcarine, which then reversed from the horizontal meridian of V2d into V3d. Similarly, the ventral upper vertical meridian representation of V1 reversed into the polar angle gradient of the upper quarterfield of V2v and onto V3v. In addition, a full hemifield of visual space for hV4 adjacent to V3v was measured in all subjects along the posterior fusiform gyrus.

**Figure 3:**
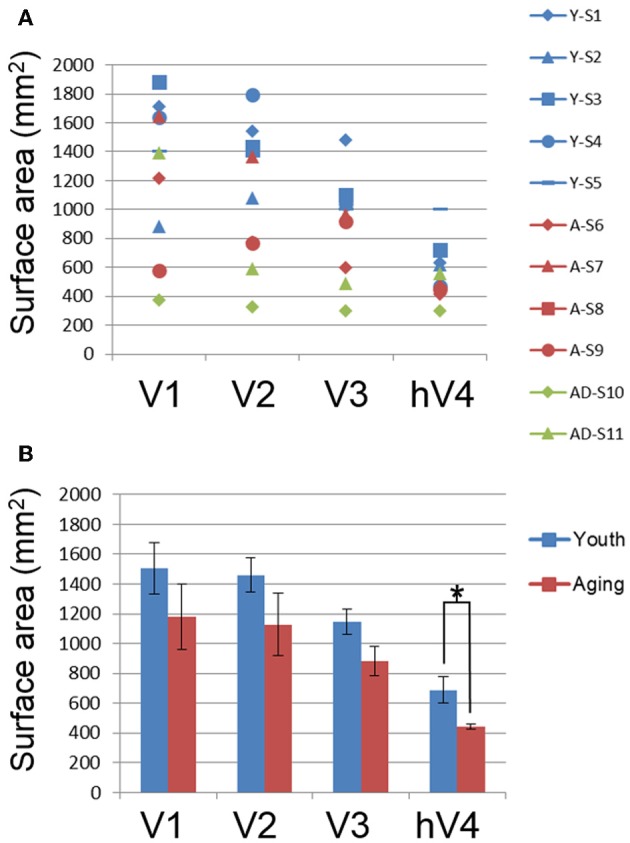
**Total surface area measurements for visual field maps**. **(A)** Total surface area for each map for individual young (blue), healthy aging (red), and mild Alzheimer's disease (green) subjects. **(B)** Total surface area for each map averaged from ~0.5 to 10° across youthful subjects (blue bars) and normal aging subjects (red bars). Error bars indicate s.e.m. “^*^” marks statistically significant differences (*p* < 0.05) between youthful and healthy aging subjects in hV4.

The total surface area of each VFM is shown for individual young (blue symbols), healthy aging (red symbols) and AD (green symbols) subjects in Figure 3A and for the group averages for individual young (blue bars) and healthy aging (red bars) in Figure 3B. To determine whether there were total surface area differences between youth and aging subjects, we performed a One-Way analysis of variance (ANOVA) for each map, V1, V2, V3, and hV4. These ANOVAs revealed no difference for V1, *F*_(7, 1)_ = 1.371, *p* = 0.280 or V2, *F*_(7, 1)_ = 2.177, *p* = 0.184. There was a marginally significant difference for V3, *F*_(7, 1)_ = 4.082, *p* = 0.083. Finally, the total surface area of hV4 was significantly different between the groups *F*_(7, 1)_ = 5.903, *p* = 0.045.

#### Aging vs. youth: surface area percent distributions across eccentricity

Cortical magnification is a general property of sensory systems that reflects sensitivity to important regions of sensory space; measuring a change in the extent of a particular part of the eccentricity gradient along cortex between subject groups would suggest differences in the functional properties of that region of cortex. While historically more common, existing estimates of the cortical magnification factor from human fMRI measurements typically only take one dimension of cortical space into account (position along eccentricity axis) and ignore the other (position along polar angle axis) (e.g., Dougherty et al., 2003). Thus, the cortical magnification factor as a function of position along the eccentricity axis does not reflect the magnification of representation along an iso-eccentricity line (i.e., across polar angles). Here we measured surface area percent distribution across the eccentricity-band ROIs to provide a measure that takes this “width” across polar angles into account.

**Figure 4:**
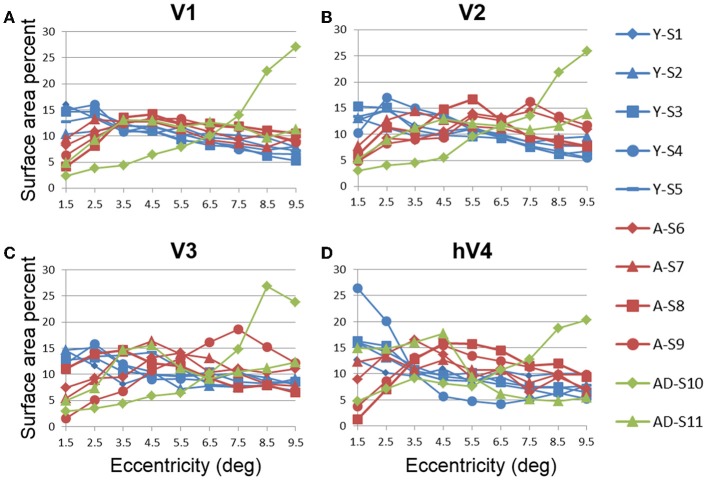
**Surface area percent distribution measurements for visual field maps in individual young, healthy aging, and mild Alzheimer's disease subjects**. **(A)** V1. **(B)** V2. **(C)** V3. **(D)** hV4. Blue lines represent data from healthy young subjects, red lines represent data from healthy aging subjects, and green lines represent data from mild AD subjects. Each line represents data measured in individual subjects and averaged across both of each subject's hemispheres. Surface area percent is plotted as a function of degrees of eccentricity from 1 to 10° eccentricity (bins centered on 1.5–9.5°). Note the consistency for both the youthful and healthy aging subjects. Note also the different distribution for AD-S10 relative to the other subjects.

**Figure 5:**
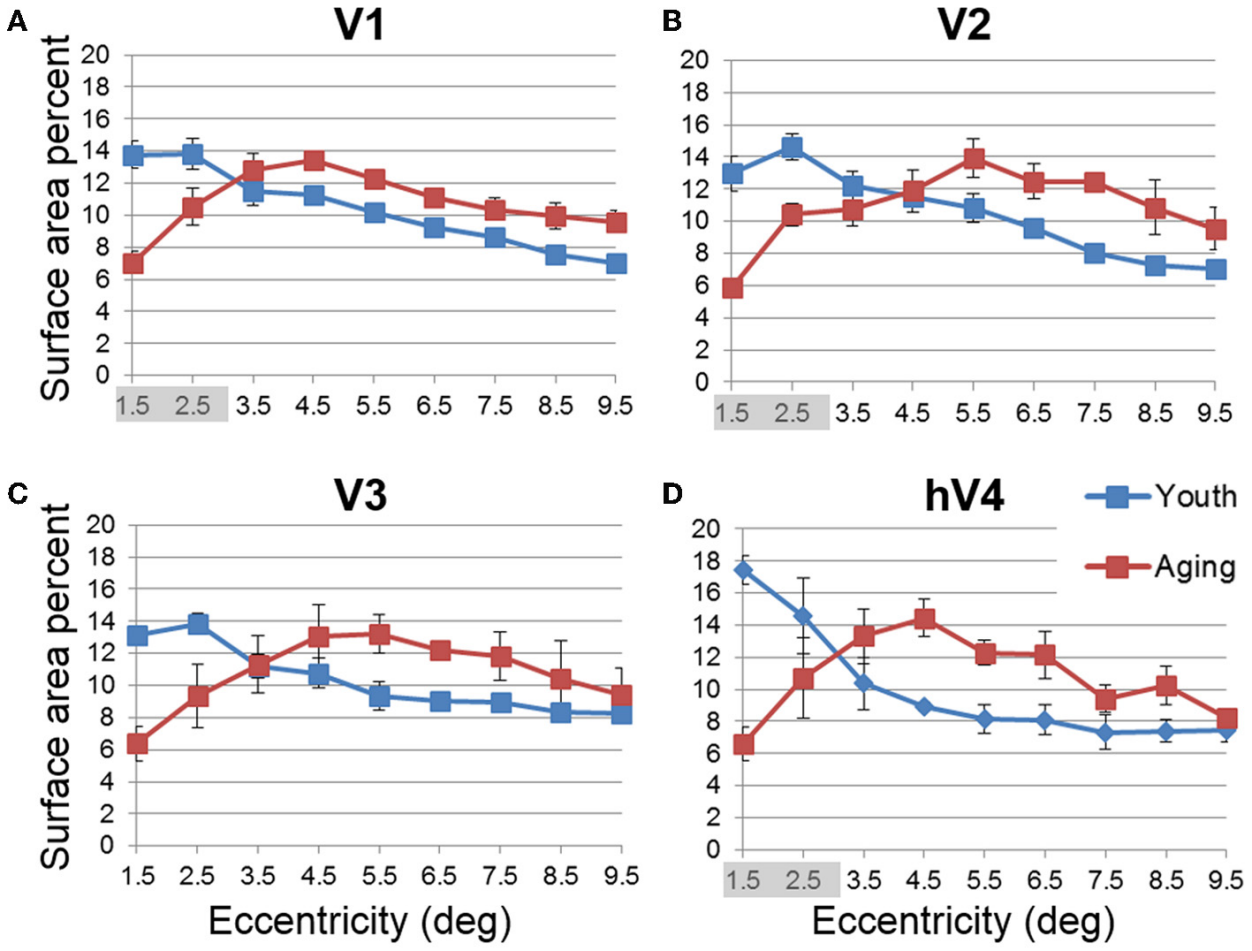
**Average surface area percent distribution measurements for visual field maps in young and healthy aging subjects**. **(A)** V1. **(B)** V2. **(C)** V3. **(D)** hV4. Blue lines represent data from healthy young subjects, and red lines represent data from healthy aging subjects. Each bar represents data measured in individual subjects and then averaged by eccentricity band across hemispheres. Surface area percent is plotted as a function of degrees of eccentricity from 1 to 10° eccentricity (bins centered on 1.5–9.5°). Shaded gray regions indicate significant differences (*p* < 0.05) between regions (0.5–3°) in each map. Note the relatively increased fovea distribution in the youthful subjects. Error bars indicate s.e.m.

In order to examine differences in VFM surface area percent distribution between the healthy aging and young subjects, we compared the average surface area percent distribution for the eccentricity-band ROIs in each VFM (Figures 4, 5, blue (young) and red (aging) lines). With the parameters (e.g., width, spatial frequency) of the moving bar stimulus, we cannot obtain clear measurements of surface area to the full center (0°) of the visual field representation nor at the very peripheral edge of the stimulus range (11°), as is typical for these stimuli (Dumoulin and Wandell, 2008). Thus, for the percent surface area measurements, we restrict the data presented in Figures 4, 5 to the 1–10° eccentricity range (i.e., the bins centered on 1.5–9.5°), allowing better comparison of these measurements to previous measures of cortical magnification with human fMRI. Although we standardly define the boundaries of VFMs on a flattened representation of cortex for optimal visualization of the polar angle and eccentricity gradients and boundaries, all ROIs are always transformed back into the 3D cortical representation to make the measurements of surface area along the 3D, folded cortical manifold to avoid the distortions induced by flattening cortex (Wandell et al., 2007; Brewer and Barton, 2012b). Overall, we observed a significant decrease (*p* < 0.05; see comparisons below) of surface area percent distribution in the central 3° for V1, V2, and hV4 between young and aging subjects, consistent with both previous measures in V1 (Crossland et al., 2008; Brewer and Barton, 2012a) and expectations from behavioral measures of decreased acuity (Elliott, 1987; Whitaker and Elliott, 1992).

***Statistical analyses***.

- V1 Comparisons: We previously reported a comparison of V1 measurements of *absolute* surface area for specific eccentricity ranges between young and healthy aging subjects in Brewer and Barton (2012a). Here we have normalized this absolute surface area by total VFM surface area and present the surface area percent distribution. A MANOVA revealed a significant decrease in surface area representing the central 3° for aging relative to youthful subjects [*F*_(3, 5)_ = 6.618, *p* = 0.034], but not the peripheral 3–10° of V1 [*F*_(7, 1)_ = 1.737, *p* = 0.527].
- V2 Comparisons: A MANOVA revealed a significant decrease in surface area representing the central 3° for aging relative to youthful subjects [*F*_(3, 5)_ =13.224, *p* = 0.008], but not the peripheral 3–10° of V2 [*F*_(7, 1)_ = 11.038, *p* = 0.224].
- V3 Comparisons: A MANOVA revealed a marginally significant decrease in surface area representing the central 3° for aging relative to youthful subjects [*F*_(3, 5)_ = 3.665, *p* = 0.098]), but not the peripheral 3–10° of V3 [*F*_(7, 1)_ = 3.297, *p* = 0.401].
- hV4 Comparisons: A MANOVA revealed a significant decrease in surface area representing the central 3° for aging relative to youthful subjects [*F*_(3, 5)_ = 11.685, *p* = 0.011], and a marginally significant decrease in the peripheral 3–10° of hV4 [*F*_(7, 1)_ = 81.063, *p* = 0.085].

#### Aging vs. youth: visual field map response variance explained

**Figure 6:**
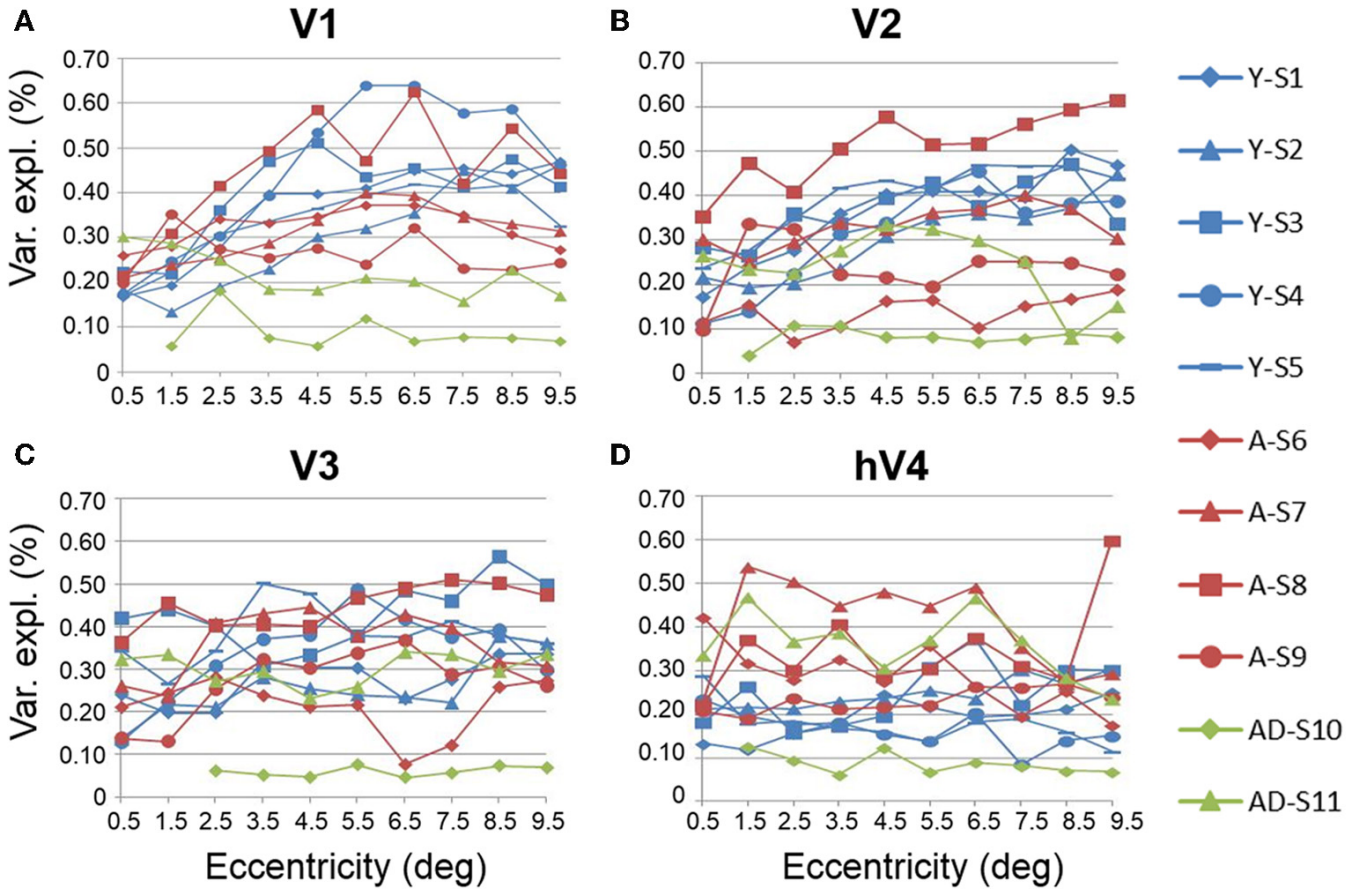
**Measurements of variance explained for visual field maps in individual young, healthy aging, and mild Alzheimer's disease subjects. (A)** V1. **(B)** V2. **(C)** V3. **(D)** hV4. Blue lines represent data from healthy young subjects, red lines represent data from healthy aging subjects, and green lines represent data from mild AD subjects. Each line represents data measured in individual subjects and averaged across both of each subject's hemispheres. Variance explained is plotted as a function of degrees of eccentricity from 0 to 10° eccentricity (bins centered on 0.5–9.5°g). Note the consistency for the youthful subjects and the somewhat greater variability for the healthy aging subjects. Note also the different distribution for AD-S10 relative to the other subjects.

**Figure 7:**
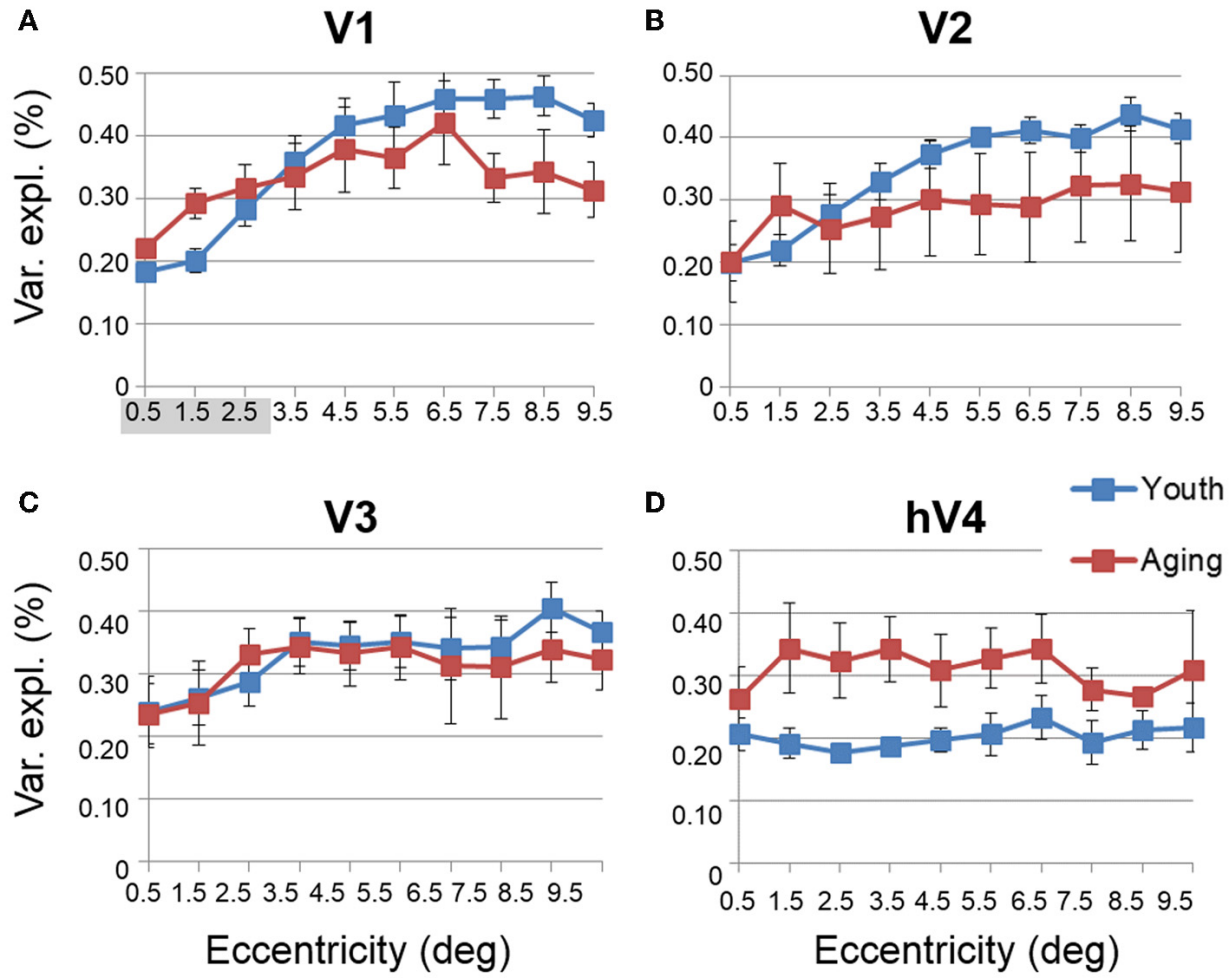
**Average measurements of percent variance explained for visual field maps in young and healthy aging subjects**. **(A)** V1. **(B)** V2. **(C)** V3. **(D)** hV4. Blue lines represent data from healthy young subjects, and red lines represent data from healthy aging subjects. Each bar represents data measured in individual subjects and then averaged by eccentricity band across hemispheres. Variance explained percent is plotted as a function of degrees of eccentricity from 0 to 10° eccentricity (bins centered on 0.5–9.5°). Shaded gray regions indicate significant differences (*p* < 0.05) between regions (0–3°) in each map. Error bars indicate s.e.m.

Another useful characterization of the functional differences between two groups is to evaluate the responsivity of particular regions of cortex. To compare the levels of BOLD activity of each VFM between healthy aging and young subjects, we measured the average variance explained of all voxels (no threshold) within each of the 10 eccentricity-band ROIs in each VFM for each subject (Figure 6, blue (young) and red (aging) lines). This average variance explained per ROIs was then averaged by ROI across each VFM within each subject group (Figure 7, blue (young) and red (aging) lines). Statistical results are again shown for each VFM in the sections below. Overall, we observed a significant increase (*p* = 0.032; see comparisons below) in the variance explained of the central 3° of V1 in aging relative to youthful subjects.

***Statistical analyses***.

- V1 Comparisons: A MANOVA revealed a significant increase in the variance explained representing the central 3° for aging relative to youthful subjects [*F*_(3, 5)_ = 6.817, *p* = 0.032], but not the peripheral 3–10° of V1 [*F*_(7, 1)_ = 0.559, *p* = 0.777].
- V2 Comparisons: A MANOVA revealed no significant change in the variance explained representing the central 3° for aging relative to youthful subjects [*F*_(3, 5)_ = 2.346, *p* = 0.190], or the peripheral 3–10° of V2 [*F*_(7, 1)_ = 0.601, *p* = 0.762].
- V3 Comparisons: A MANOVA revealed no significant change in the variance explained representing the central 3° for aging relative to youthful subjects [*F*_(3, 5)_ = 0.458, *p* = 0.723], or the peripheral 3–10° of V3 [*F*_(7, 1)_ = 0.223, *p* = 0.801].
- hV4 Comparisons: A MANOVA revealed no significant change in the variance explained representing the central 3° for aging relative to youthful subjects [*F*_(3, 5)_ = 0.350, *p* = 0.128], or the peripheral 3–10° in hV4 [*F*_(7, 1)_ = 1.832, *p* = 0.516].

#### Aging vs. youth: pRF sizes across visual field maps

**Figure 8:**
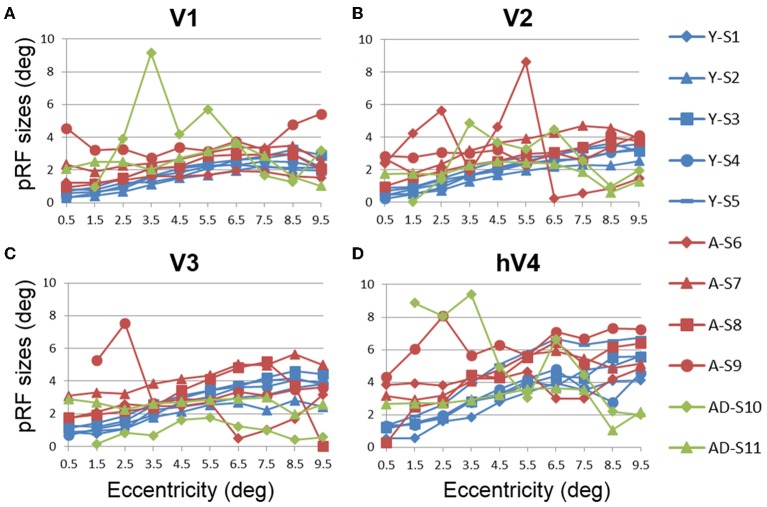
**Population receptive field size measurements for visual field maps in individual young, healthy aging, and mild Alzheimer's disease subjects. (A)** V1. **(B)** V2. **(C)** V3. **(D)** hV4. Blue lines represent data from healthy young subjects, red lines represent data from healthy aging subjects, and green lines represent data from mild AD subjects. Each line represents data measured in individual subjects and averaged across both of each subject's hemispheres. pRF size is plotted as a function of degrees of eccentricity from 0 to 10° eccentricity (bins centered on 0.5–9.5°). Note the strong consistency for the youthful subjects, whereas healthy aging subjects are generally consistent, with one subject showing large deviations for each map. Also note the differences between the two AD subjects.

**Figure 9:**
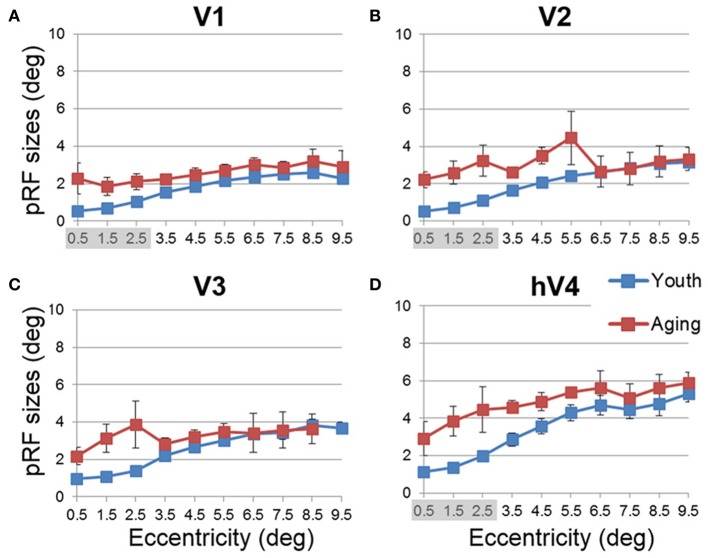
**Average population receptive field size measurements for visual field maps in young and healthy aging subjects**. **(A)** V1. **(B)** V2. **(C)** V3. **(D)** hV4. Blue lines represent data from healthy young subjects, and red lines represent data from healthy aging subjects. Each bar represents data measured in individual subjects and then averaged by eccentricity band across hemispheres. Average pRF size is plotted as a function of degrees of eccentricity from 0 to 10° eccentricity (bins centered on 0.5–9.5°). Shaded gray regions indicate significant differences (*p* < 0.05) between regions (0–3°) in each map. Error bars indicate s.e.m.

Like cortical magnification, the receptive field spread (or size) of sensory systems reflects sensitivity to important regions of sensory space, with smaller receptive fields giving a higher resolution of processing. In the healthy young human, the foveal representation is not only magnified in terms of cortical surface area, but also has the smallest receptive fields as measured with fMRI using pRF modeling (Dougherty et al., 2003; Dumoulin and Wandell, 2008). Here we also measured the sizes of pRFs (σ) as a function of eccentricity, averaged across subjects for each of the 10 eccentricity-band ROIs in each VFM (Figures 8, 9, blue (young) and red (aging) lines) and compared these measurements between healthy aging and young subjects. Statistical results are again shown for each VFM in the sections below. Generally, we observed a statistically significant (*p* < 0.05; see comparisons below) increase in pRF sizes for the central 3° in V1, V2, and hV4. These changes are also consistent with previous measures in V1 (Crossland et al., 2008; Brewer and Barton, 2012a) and expectations from behavioral measures of decreased visual acuity (Elliott, 1987; Whitaker and Elliott, 1992).

***Statistical analyses***.

- V1 Comparisons: A MANOVA revealed a significant increase in pRF size in the central 3° for aging relative to youthful subjects [*F*_(3, 5)_ = 6.285, *p* = 0.038], but not the peripheral 3–10° of V1 [*F*_(7, 1)_ = 0.932, *p* = 0.665].
- V2 Comparisons: A MANOVA revealed a significant increase in pRF size in the central 3° for aging relative to youthful subjects [*F*_(3, 5)_ = 5.869, *p* = 0.043], but not the peripheral 3–10° of V2 [*F*_(7, 1)_ = 28.343, *p* = 0.144].
- V3 Comparisons: A MANOVA revealed a marginally significant increase in pRF size in the central 3° for aging relative to youthful subjects [*F*_(3, 5)_ = 4.294, *p* = 0.075] and the peripheral 3–10° of V3 [*F*_(7, 1)_ = 89.348, *p* = 0.081].
- hV4 Comparisons: A MANOVA revealed a significant increase in pRF size in the central 3° for aging relative to youthful subjects [*F*_(3, 5)_ = 6.205, *p* = 0.039], but not the peripheral 3–10° of hV4 [*F*_(7, 1)_ = 25.680, *p* = 0.151].

### Visual field map measurements in subjects with mild alzheimer's disease

**Figure 10:**
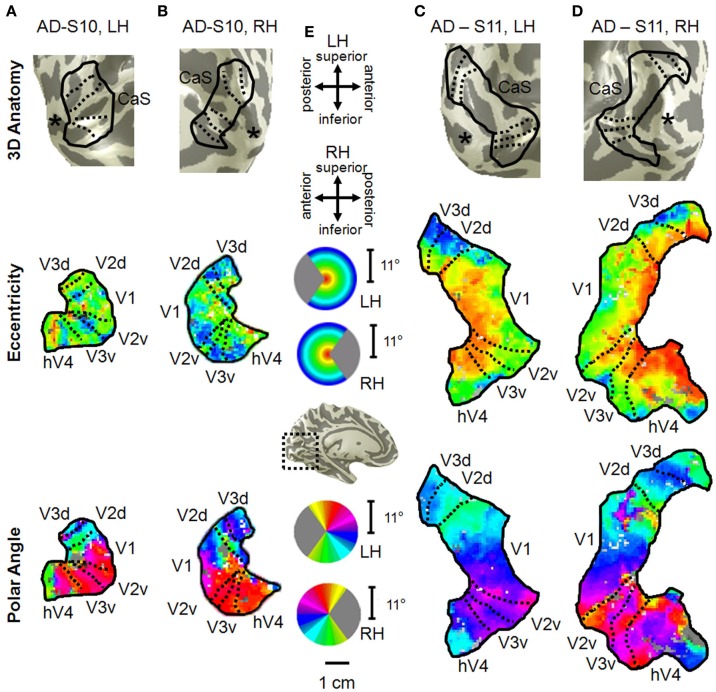
**Visual field map measurements in mild Alzheimer's disease subjects**. Occipital VFMs V1, V2, V3, and hV4 are shown for the left **(A)** and right **(B)** hemispheres of a single subject with mild AD (S10). While the polar angle gradients (bottom panel) still contained the expected representations of contralateral visual space with orderly reversals between VFMs, the eccentricity measurements (middle panel), drawn from the same fMRI scans, were more disorganized. Also note the visibly smaller size of these VFMs in this subject compared to those shown for young and healthy aging subjects in Figure 2. A second set of VFMs is also shown for the left **(C)** and right **(D)** hemispheres from a second subject (S11) with mild Alzheimer's disease. This AD subject displayed more normal VFM sizes and foveal eccentricity representations, but also has visible changes in the peripheral eccentricity representations. **(E)** Legends and scale bar. Other details are as described in Figure 2. The “^*^” denotes the occipital pole.

In contrast to the healthy aging and young subject groups, the AD subjects had more irregularities in the organization of the posterior VFMs and also differed substantially from each other (Figure 10). We describe these two AD subjects here as individual cases. In AD subject S10, all four VFMs measured here in both hemispheres (Figures 10A,B) were visibly reduced in size. Despite the smaller sizes, the polar angle representations were still regularly ordered across the region, with clearly definable boundaries of the vertical and horizontal vertical meridia between each VFM (Figures 10A,B, *lower panel*). In contrast, the eccentricity representations within these maps were extremely disordered in this subject. Figures 1A,B, *middle panel*, displays the patchy peripheral (cyan-blue) and parafoveal (yellow-orange) representations throughout each of the four VFMs. Across all four VFMs in this subject, the variance explained of the pRF model fit for these measurements was decreased compared to the measurements acquired for the other subjects (Figure 6, green diamonds). This lower variance explained may reflect noisy data arising from sources other than the neurodegenerative effects of AD. However, note that the measurements of both the eccentricity and polar angle representations were drawn from the same scan, using the moving bar stimulus with the pRF modeling method as described in **METHODS** above. Thus, changes in one representation (e.g., eccentricity) that are not seen in the other (e.g., polar angle) are unlikely to arise from general problems in that particular scan. In addition, the total surface area of each VFM is shown for individual AD subjects in Figure 3A (green diamonds). The percent surface area measurements across the eccentricity-band ROIs in this subject showed a shift in representation from the foveal regions to the relatively peripheral measurements (Figure 4, green diamonds). Finally, we observed decreases in the pRF sizes within relatively more peripheral regions of V2, V3, and hV4 of this subject (Figure 8, green diamonds).

The second AD subject, S11, had a total surface area of V1 and hV4 within the ranges of aging and young subjects, but the total surface area of V2 and V3 in this subject were similarly reduced in size to those of AD subject 10 (Figure 3A, green triangles, and Figures 10C,D). The polar angle representations in this subject were also regularly ordered across the four measured VFMs (Figures 10C,D, *lower panel*). The eccentricity representations in these VFMs in this subject contained more normal foveal representations than those seen in S-10, but were now lacking the full extent of the expected peripheral representations (Figures 10C,D, *middle panel*). The percent surface area measurements across the eccentricity-band ROIs fall within the healthy aging ranges (Figure 4, green triangles). Similarly, the measurements of variance explained in this subject were within the ranges of the healthy aging subjects for four VFMS, though slightly lower in V1 (Figure 6, green triangles). As in AD subject S-10, we also observed in S-11 decreases in the pRF sizes within relatively more peripheral regions of V2, V3, and hV4 (Figure 8, green triangles).

## Discussion

Here we have shown differences in specific structural and functional characteristics of early occipital VFMs among young, healthy aging, and AD subjects. These VFM changes may underlie many of the behavioral deficits that develop in healthy aging and AD. Measurements of disordered VFMs in some AD subjects may also be useful for improving early diagnosis of this devastating neurodegenerative disease.

### Visual field map changes in healthy aging

Previous studies in healthy aging showed no change in V1 volume (Raz et al., 2004) and no change in V1 surface area spanning the tested field of view (Crossland et al., 2008; Brewer and Barton, 2012a). Our results across the entire 10 eccentricity-band ROIs in VFMs V1, V2, and V3 are consistent with these findings, with no significant differences for total VFM surface area measured between the subject groups for any of these three VFMs (Figures 2, 3). Interestingly, the total surface area of hV4 was significantly smaller in the healthy aging subjects. These are the first measurements to our knowledge of cortical surface areas for VFMs V2, V3, and hV4 in healthy aging subjects.

#### How do our results relate to previously reported visual deficits in healthy aging?

***Decline in visual acuity***. Our measurements of the differences between subject groups demonstrated a significant decrease in the foveal surface area percent distributions of VFMs V1, V2, and hV4 for healthy aging subjects relative to young subjects, with marginally significant differences in V3 (Figures 4, 5). These aging foveal decreases are consistent with the decline in visual acuity seen normally in aging (Weale, 1975; Pitts, 1982; Gao and Hollyfield, 1992; Kline et al., 2001). Our measurements of foveal changes here are unlikely to have arisen from unstable eye position, as Crossland et al. (2008) demonstrated that aging has no effect on fixation stability, and models of improper fixation do not predict our results (Baseler et al., 2002; Levin et al., 2010). Crossland et al. (2008) further measured a similar decrease in the proportion of V1 representing the fovea. Their study first defined the surface area of V1 using the wedge stimulus (polar angle response) and then measured the proportion of voxels activated by their ring stimulus (eccentricity response) within the polar-angle defined span of V1. This produces a measure of the *extent* of activity within a VFM similar to our surface area percent distribution measurements (Figures 4, 5; Brewer and Barton, 2012a). Such a decrease in the size of the aging foveal representations seen here across multiple early VMFs and in V1 in these previous studies could lead to a loss in the resolution of cortical processing of visual information within the fovea, thus diminishing visual acuity.

Further cortical changes that may contribute to the decreased visual acuity in normal aging include the differences in pRF sizes measured across the VFMs (Figures 8, 9). We measured a significant increase in pRF sizes in the foveal representations across V1, V2, and hV4 from 0 to 3° of eccentricity, with marginally significant differences in V3. The ~2° *foveal* pRF size in the aging subjects in V1 is near that of the more *peripheral* pRF sizes (e.g., 5–7° of eccentricity) in young adults seen in this study and previously (Dumoulin and Wandell, 2008; Brewer and Barton, 2012a). The increased foveal pRF sizes in aging V2, V3, and hV4 similarly are also closer to the more peripheral pRF sizes of each of these VFMs in healthy young subjects. These increases in pRF sizes could account for the previously reported decrease in visual acuity in healthy aging (Elliott, 1987; Gao and Hollyfield, 1992; Whitaker and Elliott, 1992; Kline et al., 2001). It is possible that these increases in pRF sizes are a compensatory mechanism for the decreases in the aging foveal surface area percent distributions in these VFMs. It remains to be seen whether these changes in the pRF sizes in aging early VMFs are intrinsic to these maps or are the result of changes in other regions, such as retinal thinning or changes in feedback from higher order visual areas.

***Deficits in spatial and temporal contrast sensitivity***. Elliot et al. (Elliott, 1987; Whitaker and Elliott, 1992) demonstrated a decrease in spatial contrast sensitivity at medium and high spatial frequencies with increasing age and showed that these changes are likely due to retinal and cortical changes rather than optical changes in the eye. The broadening of pRF sizes in the aging foveae of these early occipital VFMs is consistent with these reports of decreased contrast sensitivity (Figures 8, 9). The decreases in foveal surface area percent distribution in these VFMs could also play a role in these spatial contrast sensitivity impairments (Figures 4, 5; Sloane et al., 1988; Burton et al., 1993).

In addition to changes in spatial contrast sensitivity, aging subjects have decreased temporal contrast sensitivity at intermediate and high temporal frequencies (Wright and Drasdo, 1985; Mayer et al., 1988), as well as problems with motion discrimination (Gilmore et al., 1992; Wojciechowski et al., 1995). As human V1 and V3 have been implicated in motion processing (McKeefry et al., 1997; Smith et al., 1998), the significant and marginally significant decreases in foveal surface area percent distributions and increased pRF sizes of aging V1 and V3, respectively, may similarly play a role in these temporal contrast sensitivity and motion discrimination deficits (Figures 5A,C, 9A,C).

***Changes in spatial attention***. Deficits in spatial attention have been proposed to contribute to the shrinkage of the useful field of view in aging (Haas et al., 1986; Johnson et al., 1989; Haegerstrom-Portnoy et al., 1999). Measurements in macaque and human visual areas V1, V2, and V4 have demonstrated neural mechanisms possibly subserving selective spatial attention (Luck et al., 1997; Gallant et al., 2000; Reynolds et al., 2000; Serences and Yantis, 2006). Here we observe response patterns across the V1, V2, and hV4 VFMs that could similarly contribute to deficits in spatial attention, including the significantly smaller surface area percent distributions in the fovea of these VFMs (Figures 5A,B,D). In addition, the total surface area of hV4 was decreased in the aging subjects, and V2 and hV4 both showed increases in pRF sizes across larger foveal and parafoveal regions (Figures 9B,D), all of which could reflect deficits in the proper tuning of spatial attention.

***Color vision deficiencies***. Finally, aging subjects frequently demonstrate losses in color discrimination, especially along the blue-yellow axis, much of which can be attributed to changes in the aging lens (Haegerstrom-Portnoy et al., 1988; Johnson et al., 1988; Haegerstrom-Portnoy et al., 1999; Bron et al., 2000). However, concurrent or consequential neural changes have not been ruled out. Here we note significant differences in pRF sizes for 0 to 3° in V1, V2, and hV4 (Figures 9A,B,D). In addition, our measurements showed both a decrease in total surface area in hV4 and an *increase* in the BOLD variance explained over the central 0 to 3° of eccentricity in V1 in healthy aging relative to youthful subjects (Figures 1B, 7A). It is possible that these expanded pRFs in aging subjects are associated with aging changes specific to a ventral visual color and form pathway involving V1, V2, and hV4. Also, similar increases in occipital activity in healthy aging subjects in studies of visual working memory have been suggested to be a sign of some form of compensatory cognitive activity (Alichniewicz et al., 2012), which could be playing a role here.

### Visual field map changes in mild alzheimer's disease

Our measurements in mild AD subjects here both demonstrate the feasibility of making these VFM measurements in patients with dementia and highlight the need for such detailed analyses in individual subjects for these types of investigations. Each subject differed in the changes in the overall organization of these VFMs, with one subject (S10) having visibly small, disordered VFMs with low variance explained across the medial occipital surface and one (S11) having grossly normal VFM organization (Figure 2). These differences are likely due to variations in the pattern and progression of neurodegeneration in each subject. Even so, there are patterns of changes across these four hemispheres that may reflect more uniform effects of AD on the visual pathways. Such changes may underlie the visual symptoms seen early in the disease (Katz and Rimmer, 1989) and may prove to be a useful tool for early and accurate diagnosis of AD.

#### Potential for improvement of dementia diagnosis

One of the goals in optimizing the diagnosis of dementia is to detect cortical changes very early with the hope that early intervention can lead to more effective treatments that stop the progression of dementia before much irreversible cortical damage ensues (Rosen, 2004). Recent research in early diagnosis spans cognitive testing to biochemical markers to neuroimaging methods such as positron emission tomography (PET), structural MRI, and functional MRI (Graham et al., 2004; Naggara et al., 2006; Ringman et al., 2008). Our neuroimaging results here in subjects soon after a diagnosis of mild AD open up the possibility of the use of detailed VFM measurements for early diagnosis as well. Individual subject VFM analysis allows both for these detailed measurements and for the ability to track these changes in specific individuals over time. Because VFMs are highly-structured functional responses in cortex that can be measured non-invasively with fMRI, they may prove useful for demonstrating very subtle changes early in AD. Future studies should expand upon our findings in AD with a broader range of AD subjects as well as measurements of the development of visual symptoms in patients with mild cognitive impairment (MCI; Mapstone et al., 2003; Tabert et al., 2006; Alichniewicz et al., 2012).

Disagreement also persists in the categorization of neurodegenerative symptoms into specific types of dementia. Criteria have been outlined to differentiate AD from other dementias (e.g., Dementia with Lewy Bodies, Posterior Cortical Atrophy), but there still remains significant overlap across the symptoms associated with each dementia (e.g., Harding et al., 2002; Tang-Wai et al., 2004; Armstrong et al., 2005; Sauer et al., 2006). The differences in the initiation of the neuropathology of the various types of dementia are not well understood, but these differences may be important to the types of treatment required for each specific dementia. Highly detailed measurements of specific changes within VFMs such as those we present here may be able to provide distinctive differences between types of dementia, which likely have different patterns in the onset and severity of visual symptoms.

#### Patterns of neurodegeneration in the visual cortex of AD subjects

Our measurements here in 2 mild AD subjects provide the initial steps toward reaching this goal of improved diagnosis. Our results in these first subjects show that such measurements are possible in this patient population and suggest a combination of patterns of neurodegeneration, with specific changes in cortical representations (Figures 3, 4, 6, 8) in addition to differences in gross VFM organization (Figure 10). Measurements of both aspects of distributed neurodegeneration in the visual pathways may be useful in the diagnosis of AD in a specific individual and for understanding the progression this disease across cortex.

Visual deficits often reported as one of the first symptoms of AD include problems with visual attention, visual processing speed, visual field defects, contrast sensitivity, color discrimination, visuospatial processing, and feature recognition of complex objects such as faces (Parasuraman et al., 1992; Cronin-Golomb et al., 1993; Giannakopoulos et al., 1999; Chan et al., 2001; Holroyd and Shepherd, 2001; Jackson and Owsley, 2003; Mapstone et al., 2003; Tang-Wai et al., 2004; Thiyagesh et al., 2009). Interestingly, Subject S10 had very disorganized eccentricity maps with little foveal representation, possibly due to an idiosyncratic pattern of neurodegeneration around the occipital pole, although these eccentricity measurements may be complicated by the low variance explained of the pRF fit for these measurements (Figures 10A,B). Patients with such foveal loss might present with the deficiencies in color and form processing frequently described in AD (Cronin-Golomb et al., 1993; Chan et al., 2001; Sauer et al., 2006). These changes in VFMs could arise either from bottom-up effects from degenerative disease in the retina and optic nerves or from top-down changes in feedback from higher order visual areas, which have been shown in some studies to have a greater lesion load in AD than primary visual cortex (Lewis et al., 1987; Jackson and Owsley, 2003). Overall, our first characterization of these VFMs in AD subjects shows intriguing individual differences in the neurodegenerative patterns affecting visual cortex and emphasizes the need for additional studies of the timing and extent of VFM alterations in a large population of AD patients.

## Conclusions

Our measurements first investigate whether there is a systematic change in visual cortex as part of the normal aging process Such knowledge of how visual representations change with healthy aging will allow us to explore both the effects of normal aging on the perceptual system and improve our ability to use age-matched controls in studies of age-related diseases (Jackson and Owsley, 2003; Yankner et al., 2008). We then demonstrate the feasibility and first characterization of these measurements in patients with mild AD. Our hope is that such data will contribute to earlier and more definitive detection of these forms of dementia and a better understanding of the differences between AD and other dementias.

### Conflict of interest statement

The authors declare that the research was conducted in the absence of any commercial or financial relationships that could be construed as a potential conflict of interest.
